# Assessment of the Corrosion Rate of Maraging Steel M350 Produced by Additive Manufacturing Using the Laser Powder-Bed Fusion Method and Surface Finishing Techniques

**DOI:** 10.3390/ma18174098

**Published:** 2025-09-01

**Authors:** Krzysztof Żaba, Martyna Szczepańska, Maciej Balcerzak, Sławomir Kac, Piotr Żabinski

**Affiliations:** 1 Department of Metal Working and Physical Metallurgy of Non-Ferrous Metals, Faculty of Non-Ferrous Metals, AGH University of Krakow, al. Adama Mickiewicza 30, 30-059 Krakow, Poland; krzyzaba@agh.edu.pl (K.Ż.); maszczep@student.agh.edu.pl (M.S.); balcerzak@agh.edu.pl (M.B.); 2 Faculty of Metals Engineering and Industrial Computer Science, AGH University of Krakow, al. Adama Mickiewicza 30, 30-059 Krakow, Poland; kac@agh.edu.pl; 3 Department of Physical Chemistry and Metallurgy of Non-Ferrous Metals, Faculty of Non-Ferrous Metals, AGH University of Krakow, al. Adama Mickiewicza 30, 30-059 Krakow, Poland

**Keywords:** M350 steel, corrosion, surface treatment, 3D printing, additive manufacturing

## Abstract

The objective of this study was to investigate the influence of additive manufacturing parameters, specifically using laser powder bed fusion (LPBF), and surface finishing methods on the corrosion rate and behavior of maraging steel M350 components. Samples were fabricated via LPBF employing varying laser powers (80 W, 100 W, and 120 W) and subsequently subjected to mechanical polishing. Corrosion performance was evaluated through 450 h immersion tests in a 3.5% aqueous NaCl solution and potentiodynamic polarization measurements. Microstructural characterization and surface topography assessments were performed using optical microscopy, scanning electron microscopy coupled with energy-dispersive spectroscopy (SEM-EDS), and profilometry. The results demonstrate a strong influence of temperature, manufacturing parameters, and polishing on corrosion processes. At room temperature, higher laser power reduced corrosion rates due to better powder consolidation and lower porosity, whereas at 45 °C, the trend reversed, with the highest corrosion rates observed for samples produced at 120 W. Mechanical polishing significantly reduced surface roughness (Ra from ~7–10 μm to ~0.6–1 μm) but did not improve corrosion resistance; in some cases, it increased corrosion rates, likely due to stress redistribution and exposure of subsurface defects. Potentiodynamic tests confirmed that higher laser power reduced corrosion current density for unpolished surfaces, but polishing increased current density at 80 W more than twofold. The findings indicate that optimizing LPBF process parameters is crucial for improving the corrosion resistance of M350 steel. High laser power (≥120 W) is beneficial at ambient conditions, while lower powers (80–100 W) perform better at elevated temperatures. Mechanical polishing alone is insufficient for enhancing resistance and should be combined with stress-relief and porosity-reduction treatments. These results provide guidelines for tailoring additive manufacturing strategies to ensure reliable performance of M350 steel in chloride-rich environments.

## 1. Introduction

The use of additive manufacturing technologies, particularly metal 3D printing, is gaining increasing popularity in industry due to the growing availability of advanced equipment capable of producing components using methods such as directed energy deposition (DED) and laser powder bed fusion (LPBF) [1,2]. This trend is primarily driven by the rising demand for manufacturing techniques that enable the rapid and precise production of complex assemblies and single components [3]. These technologies are based on the selective melting of metal powder using a concentrated energy source, typically a laser, or in some cases an electron beam [4,5]. Additive manufacturing facilitates the fabrication of advanced components that are often impossible to produce using conventional machining methods such as turning or milling [6,7]. These techniques are especially advantageous in the production of prototypes or small production batches due to significantly lower costs resulting from simplified manufacturing processes and a reduced number of technological operations required to obtain a finished part [8]. Although metal 3D printing remains relatively expensive, it is most commonly applied in the production of components that demand high mechanical performance and precision [9]. In the context of prototyping, additive manufacturing allows for design modifications with minimal financial investment, as the effort and cost required to produce several different components are comparable to those needed for producing multiple identical parts [10].

Despite their numerous advantages, additive manufacturing methods also present significant limitations. High unit costs and long production times make them unsuitable for mass production. Several economic factors influence the cost-effectiveness of 3D printing, including the price of the metal powder, the efficiency of the printing equipment, and the cost of shielding gases used during the process. Many companies are increasingly adopting additive manufacturing technologies due to their ability to minimize material waste and streamline the supply chain. A wide range of additive manufacturing systems and technologies are currently available on the market, with the most prominent including selective laser melting (SLM), direct metal laser sintering (DMLS), electron beam melting (EBM), binder jetting, directed energy deposition (DED), and laser powder bed fusion (LPBF). These techniques vary in terms of process parameters, component fabrication mechanisms, production time, dimensional accuracy, and the mechanical properties of the final parts. Research efforts in the field of metal additive manufacturing are primarily focused on optimizing process parameters and verifying the quality of the printed parts [11,12]. Key parameters subject to optimization include laser power, layer thickness, and scan speed, as they significantly affect the mechanical properties and density of the printed material [13,14]. To improve dimensional accuracy and surface quality, studies are also being conducted on hybrid manufacturing techniques that combine additive manufacturing with subtractive machining processes [15]. Another important research direction involves reducing part weight and enhancing performance by leveraging the design flexibility offered by additive manufacturing [16,17]. Researchers have also examined the potential for additive manufacturing to support sustainable development by reducing CO_2_ emissions and minimizing material consumption [18,19]. A notable area of focus involves the development of novel powder materials and the refinement of process parameters to enable their effective use in additive manufacturing [20,21,22]. Furthermore, the integration of additive technologies with digital manufacturing systems is being explored as a means of accelerating the product development cycle and reducing time to market [23,24].

A wide variety of metal alloys are used in 3D printing technologies, with the most common being steels, nickel-based alloys, aluminum alloys, and titanium alloys. Among steels, maraging steels are particularly popular due to the high strength of the printed components, excellent weldability, and dimensional stability [25,26]. Maraging steels can be classified into several variants, including maraging 200, 250, 300, 350, and 400, which differ in terms of chemical composition and mechanical properties [27]. The primary alloying elements in this group of steels include nickel (13–19%), cobalt (7–15%), molybdenum (3–10%), and titanium (0.2–1.6%) [28]. The most commonly used grade in 3D printing is maraging steel M300. According to powder manufacturers, its typical composition includes 17–19% nickel, 7–9% cobalt, 4.5–5.2% molybdenum, and 0.3–1.2% titanium [28,29]. The widespread use of this alloy is attributed to its availability, excellent mechanical properties, ease of heat treatment, good weldability with minimal shrinkage, high machinability in the soft state, and dimensional stability [30,31]. One of its major advantages in additive manufacturing is that it is hardened through solution annealing followed by aging, rather than the conventional quenching used for traditional steels [32,33,34,35].

In the present study, maraging steel 18Ni-350 (M350) was employed. It is one of the most widely used grades in this class of materials and is characterized by elevated levels of cobalt (11.5–12.5%) and titanium (1.3–1.6%), which significantly enhance mechanical strength and thermal stability under varying strain rates and temperature fluctuations [36,37]. Following solution annealing and aging, M350 achieves a yield strength in the range of 1900–2400 MPa and a hardness of approximately 54–58 HRC [38,39]. Its elongation at fracture is about 10%, indicating a favorable balance between strength and ductility [39]. These properties make M350 a material of choice for aerospace, defense, automotive, and tooling applications [36,40]. Research on the use of M350 maraging steel in 3D printing processes—such as laser powder bed fusion (LPBF) and selective laser melting (SLM)—is ongoing, with particular emphasis on process parameter optimization and evaluation of the mechanical and microstructural properties of printed parts [41]. Researchers focus especially on the influence of laser power, layer thickness, and scan speed on the quality of M350 prints. Studies have demonstrated that proper parameter selection can substantially improve mechanical performance, including impact resistance [42].

M350 is a high-grade martensitic stainless tool steel characterized by a balanced combination of mechanical strength, machinability, polishability, and moderate corrosion resistance. Owing to its chemical composition, which includes approximately 13.5% chromium along with molybdenum and vanadium, M350 exhibits substantially improved corrosion resistance compared to conventional tool steels such as D2 and H13. Although it does not achieve the corrosion resistance levels of austenitic stainless steels like AISI 304 or 316, it demonstrates satisfactory performance in moderately corrosive environments, particularly in applications such as plastic injection molding where exposure to moisture or water-based cooling systems is common. Nevertheless, its use is not recommended in highly aggressive environments, such as marine settings or in contact with concentrated acids. Optimal corrosion resistance can be attained through appropriate heat treatment and surface finishing techniques, including polishing and passivation.

This research focuses on evaluating the corrosion behavior of 3D-printed M350 steel components with two distinct surface finishes. The samples were immersed in oxygen-rich, chloride-containing water at both room temperature and elevated temperatures. To determine the corrosion rate, electrochemical polarization tests and weight loss measurements were performed. Additionally, the corroded specimens were examined microscopically to better understand the corrosion processes and to explore how surface finish and microstructural features at the material interface influence corrosion resistance.

## 2. Materials and Methods

Maraging steel powder (18Ni-350, 1.6356), supplied by Rosswag GmbH (Pfinztal, Germany), was used to produce the samples using the selective laser melting printing method. The chemical composition of the powder is provided in Table 1.

An SLM280 printer from Nikon SLM Solutions AG (Lübeck, Germany) was used for additive printing of the steel samples. Table 2 lists the technical parameters of the device.

**Figure 1:**
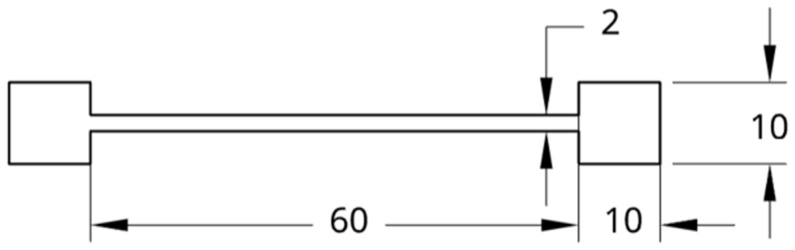
Dimensions of profiles printed using the SLM280 from Nikon SLM Solutions.

The samples were printed with the dimensions shown in Figure 1. The corroded area is 10 mm by 10 mm, with a thickness of 2 mm. The total build height (printing depth in the build direction) of the manufactured parts was 2 mm, which corresponds to the final thickness of the samples after the LPBF process. This value represents the overall dimension of the parts along the Z-axis, i.e., the direction in which layers were successively deposited. The layer thickness used during the LPBF process was 100 µm, meaning that approximately 20 layers were required to achieve the final part height. These parameters ensured stable melting conditions and uniform consolidation of the material throughout the build.

After 3D printing, all samples were cleaned with distilled water and then with ethanol and allowed to air dry at room temperature. Additionally, some of the prints were mechanically polished. Before further testing, the samples were cut and halved.

The steel samples were subjected to the following corrosion tests:

- Long-term corrosion test—measuring the mass change before and after a 450 h period of immersion in a 3.5% aqueous NaCl solution;
- Potentiodynamic polarization test.

The tests were conducted on M350 maraging steel samples that had been additively printed using various laser powers (80 W, 100 W, 120 W). Some samples were additionally surface-treated with mechanical polishing.

The necessary mass measurements for the long-term corrosion test were performed using a RADWAG AS 60/220.R2 analytical balance (Radwag, Radom, Poland), which has a measurement accuracy of 0.00001 g. The samples were weighed before immersion in the NaCl environment, then half of the immersed samples were placed at 45 °C, and the other half were left at room temperature (21 °C). After 450 h, the samples were removed from the containers, cleaned with distilled water and alcohol, and dried. The mass measurements were repeated, and the obtained mass results were compared to determine the effect of the test conditions on corrosion and to assess the corrosion rate Vc. The following formula was used for calculations:

$$V_{c}=\frac{\Delta m}{S\;\cdot t}\;[g/cm^{2}\cdot year]$$

where V_c_ is the corrosion rate, defined as the loss of one gram of metal per square centimeter of surface area per year; Δm is the difference in sample mass before and after the corrosion test (g); S is the sample surface area (cm^2^); and t is the duration of the corrosion test (year).

Corrosion of M350 maraging steel in a 3.5% NaCl solution is an example of electrochemical corrosion in which anodic and cathodic reactions occur simultaneously. The heterogeneity of the chemical composition on the surface obtained through additive manufacturing, the presence of grain boundaries, as well as the characteristic porosity, microcracks, and roughness of the upskin layers of the prints create conditions for the formation of cathodic and anodic systems in micro-areas.

The electrolyte was a non-deoxygenated NaCl solution. The presence of chlorides (Cl^−^) in the solution accelerates corrosion because Cl^−^ ions can penetrate the passive layer on the steel surface, initiating local pitting corrosion. The simplified corrosion mechanism that occurs under the conditions described above takes into account the anodic, cathodic, and secondary reactions that occur during a long-term test:

(a) Anodic reaction (metal oxidation)

Iron (Fe) oxidizes on the surface immersed in the NaCl solution, releasing it into solution as Fe^2+^ ions:

Fe → Fe^2+^ + 2e^−^

(b) Cathodic reaction (oxygen reduction)

The presence of air in the oxygenated NaCl solution causes reduction by a reaction between oxygen and water and electrons, forming hydroxide ions (OH^−^):

O_2_ + 2H_2_O + 4e^−^ → 4OH^−^

(c) Secondary reaction (formation of corrosion products)

Fe^2+^ ions formed in the anodic reaction react with OH^−^ ions formed in the cathodic oxide,

Fe^2+^ + 2OH^−^ → Fe(OH)_2_

Iron(II) hydroxide is unstable in the presence of oxygen and is further oxidized to iron(III) hydroxide:

4Fe(OH)_2_ + O_2_ + 2H_2_O → 4Fe(OH)_3_

The final product and main component of rust is iron(III) hydroxide, Fe(OH)_3_.

Potentiodynamic polarization tests were performed on samples with both polished and unpolished surfaces, produced using various laser power levels (80 W, 100 W, 120 W). A BioLogic SP-200 potentiostat with EC-Lab V10.44 software was used for the tests. The experiment employed a linear potential sweep of −2 V to +2 V relative to the open circuit potential (OCP), with a potential change rate of 0.01 V/s. The measurement was conducted in a vessel containing 3.5% NaCl solution. A calomel electrode served as the reference electrode, a platinum plate at least ten times larger than the surface of the test samples served as the counter electrode, and the test samples served as the working electrode.

A Nikon Elipse LV150 optical microscope and a JCM-6000 PLUS scanning electron microscope with a JED-2300 EDS detector from JEOL were used to examine the microstructure. Area EDS analysis was performed to determine the chemical composition of the observed micro-areas.

**Figure 2:**
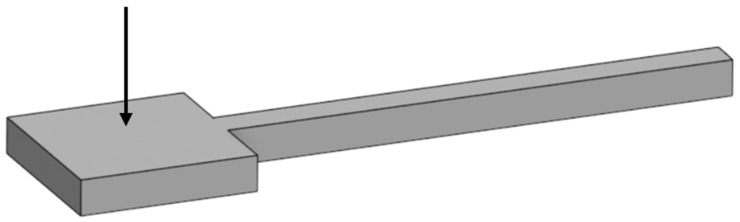
Sample surface examined with a confocal microscope.

A Wyko NT9300 optical profilometer from Veeco was used to analyze the topography and surface roughness. The study examined mechanically unpolished profiles, produced additively using laser power levels of 80 W, 100 W, and 120 W, respectively, and, for comparison, a printed, mechanically polished profile. The surfaces of the samples after long-term corrosion were also examined—both mechanically polished and unpolished. The analysis was performed at two points on the top surface of the samples (Figure 2). The measurements yielded the parameters Rz, the maximum roughness height, and Ra, the arithmetic mean deviation.

To obtain statistical representation of measurements, all experiments were repeated at least three times. Each time we used a fresh sample. This means that 58 parallel samples were used for the experimental part. All of them were printed at once using an LPBF printer and one loading of metallic powder.

## 3. Results

**Figure 3:**
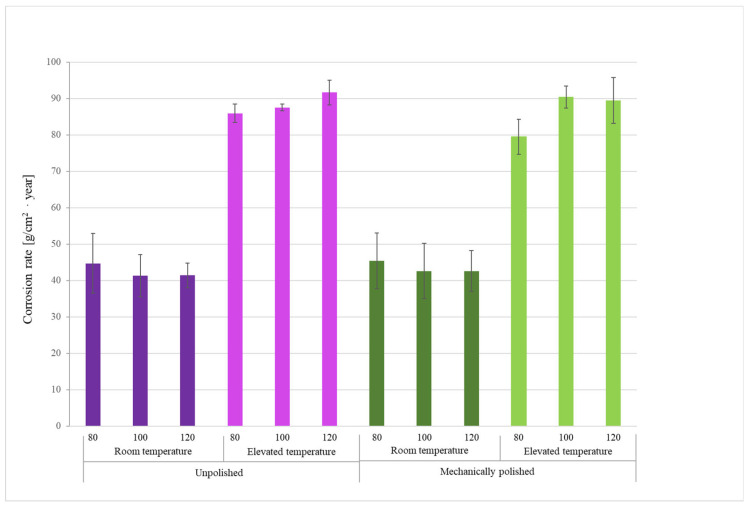
Graph of the relationship between corrosion rate and manufacturing parameters and corrosion temperature.

The long-term corrosion test was conducted for 450 h, and the average corrosion rate was determined based on measurements of the mass change of the samples before and after exposure to the corrosive environment. The measurement results are presented in Table 3 and Figure 3.

For both groups of samples—unpolished and polished—a significant increase in corrosion rate was clearly observed with increasing environmental temperature from room temperature (21 °C) to elevated temperature (45 °C). For unpolished samples produced at a laser power of 80 W, the corrosion rate increased from 44.69 g/cm^2^·year at room temperature to 85.90 g/cm^2^·year at 45 °C, representing an almost twofold acceleration of the process. A similar relationship was observed for polished samples—for example, at a power of 100 W, the corrosion rate increased from 42.56 g/cm^2^·year to 90.38 g/cm^2^·year.

In most of the analyzed series, mechanically polished samples exhibited a higher corrosion rate compared to unpolished samples. At 100 W at room temperature, the corrosion rate increased from 41.33 g/cm^2^·year (unpolished) to 42.56 g/cm^2^·year (polished). A similar relationship was observed for the remaining laser powers. Although the differences are small, they demonstrate a consistent trend—a smooth surface does not limit the number of active reaction sites and corrosion initiation at low temperatures. At an elevated temperature of 45 °C, the corrosion rate for samples produced at 100 W increased from 87.51 g/cm^2^·year to 90.38 g/cm^2^·year for polished samples, but a decrease in the corrosion rate was observed for 80 W and 120 W.

Temperature plays a key role in the corrosion process. The results of the long-term corrosion study indicate that at room temperature (21 °C), the mass loss of the tested samples was lower than at 45 °C. An increase in mass loss was observed with increasing temperature, confirming that corrosion reactions occur more rapidly at higher temperatures. This is due to the acceleration of electrochemical reactions and the increased mobility of ions in solution.

Considering the effect of additive manufacturing parameters on the obtained results, for unpolished samples, the differences between the powers of 80 W, 100 W, and 120 W are ambiguous. At room temperature, the corrosion rate decreases slightly with increasing power: from 44.69 g/cm^2^·year for 80 W to 41.39 g/cm^2^·year for 120 W.

This suggests that higher laser power, due to improved powder remelting and reduced porosity, improves the structure quality, which limits electrolyte penetration. For samples at 45 °C, the opposite phenomenon is observed: the higher the laser power, the faster the corrosion rate and the lower the material’s corrosion resistance. For polished samples, increasing laser power also leads to a slight decrease in the corrosion rate at room temperature, but at 45 °C, the highest values are observed for 100 and 120 W. This may be due to the combined effect of increased printing process energy and high test temperature, leading to local overheating of the structure and weakening of the passive layer.

**Figure 4:**
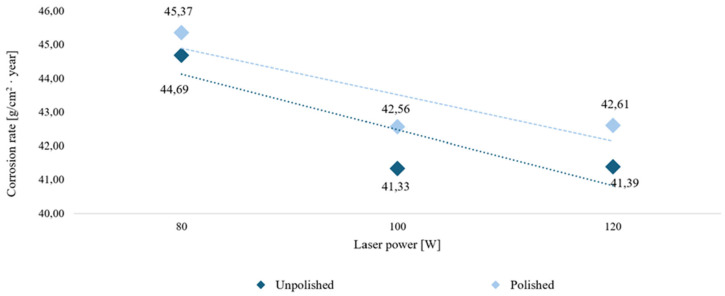
Corrosion rate trend lines in a long-term room temperature test on unpolished and polished surfaces.

**Figure 5:**
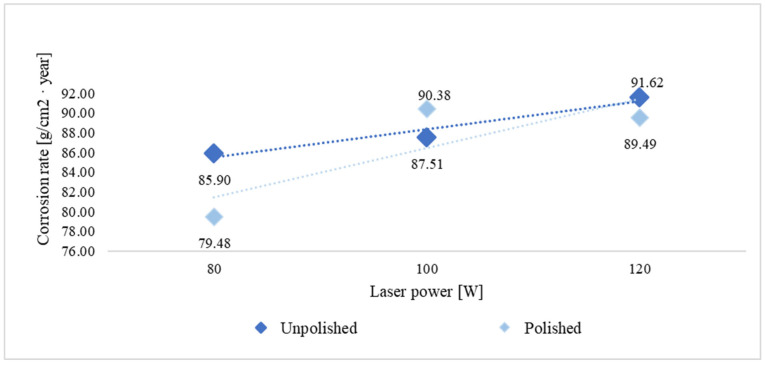
Corrosion rate trend lines in a long-term elevated temperature test on unpolished and polished surfaces.

The graphs in Figure 4 and Figure 5 show the trend in corrosion rate depending on the cited variable parameters for the long-term study. The graphs show a clear trend for the two test temperature groups. It was observed that at room temperature, with increasing laser power, the corrosion rate decreases, regardless of the surface treatment. However, at elevated temperatures, the use of higher laser power in the manufacturing process results in poorer corrosion resistance, as the corrosion rate increases with increasing voltage. In summary, M350 maraging steel produced with higher laser power is more suitable at lower temperatures, while at elevated temperatures, lower laser power can be used. However, surface treatment can further enhance corrosion resistance at elevated temperatures.

Surface treatment has a significant impact on the corrosion resistance of the tested samples. An uneven, rough surface increases the area of contact with the corrosive solution, accelerating the degradation process. Mechanical polishing, which leads to a smooth surface, reduces the area exposed to corrosive agents and eliminates defects that could initiate corrosion. However, mechanical processing can also introduce stresses, and when these stresses are high, they can weaken the passive layer and accelerate corrosion, especially in the presence of aggressive agents. Furthermore, it is worth noting that additively manufactured surfaces are characterized by the presence of localized internal stresses, such as:

- Tensile stresses, most often located in near-surface layers, resulting from rapid heating and cooling of the metal, where the temperature gradient in the melted and upskin zones causes the outer areas to shrink faster than the print core;
- Compressive stresses, present in deeper layers of the print due to compensation for surface tensile stresses, which can cause certain zones to compress;
- Multiaxial and gradient stresses, the pattern of which is non-uniform and changes with depth, creating areas of adjacent complex zones. These zones consist of tensile and compressive stresses, built up by residual stresses from expansion and contraction during the melting of successive powder layers.

These stresses can be released or transferred to deeper zones of the print by mechanical treatment such as surface polishing. Removing a thin layer of material from the upskin area, where stresses are often highest due to rapid cooling and the lack of relaxation, changes the stress equilibrium conditions. Tensile micro-stresses then transfer to the boundary between the polished zone and the print core, altering the stress gradient. Due to the observed corrosion behavior of additively manufactured M350 maraging steel, this factor should be considered in the analysis of the long-term study results.

The obtained results of mass loss and corrosion rate measurements did not indicate the expected significant improvement in the material’s corrosion resistance after mechanical surface polishing. Moreover, the obtained corrosion rate values are higher for polished samples, which makes it difficult to clearly link the effectiveness of the applied treatment with the actual behavior of the material. Therefore, the factor of evolving internal stresses can be distinguished as an independent parameter with a significant impact on the final corrosion rate values.

During the analysis of the obtained results, the dependence of the slope coefficient of the trend line curve on the influence of laser power, surface treatment, and test temperature was determined. The results are presented in Table 4.

The room temperature environment is characterized by a clear decreasing trend in average corrosion rates with increasing laser power, regardless of the sample surface condition. Both unpolished and polished samples have a negative slope coefficient, equal to −1.65 for unmachined surfaces and −1.38 for machined surfaces. Increasing laser power during the manufacturing process has a positive effect on the material’s corrosion resistance in room-temperature applications. Furthermore, the use of polishing further reduced the material’s corrosion resistance at the same temperature amplitude.

For elevated temperatures, an inverse relationship was observed: increasing laser power during generation resulted in an increase in corrosion rate. This linear trend is described by curves with slopes of 2.86 for unpolished samples and 5.01 for polished samples. The higher the temperature, the faster the ion diffusion, and therefore the more intense the electrochemical reactions. The elevated temperature of the test environment led to the degradation of protective surface layers or the increased development of microcracks and stress zones. This relationship is particularly pronounced for polished samples. Although higher power leads to better melting of the metal powder in the additive process, resulting in reduced macroscopic porosity, it can also promote a more uniform, dispersed general corrosion mechanism. Smoothing the surface reduces the number of microcracks, pores, and exposed unmelted powder, which can act as foci of local pitting corrosion. The surface is less rough and more uniform, so the aggressive corrosion environment can act simultaneously on the entire machined zone. This observation is particularly confirmed by the visual appearance of polished samples subjected to long-term corrosion at elevated temperatures.

In addition to quantitative assessments, such as mass loss and corrosion rate determination, macroscopic analysis of the sample surface is an important complement to corrosion resistance testing for a given material. Visual observation allows for the assessment of the uniformity of degradation processes and also provides a deeper perspective on the potential identification of corrosion products based on the colors of compounds deposited in the tested area. Table 5 summarizes the surface appearance of the samples before and after long-term corrosion testing, taking into account the surface treatment, test temperature, and laser power used in the sample production process.

The observed samples, regardless of the laser power or surface treatment method used, exhibited asymmetric blackening, limited to a specific surface area. These areas were noticeably darker, partially covered with a dull, dark brown deposit, indicating the intense accumulation of corrosion products, likely containing iron oxides and hydroxides—primarily Fe(OH)_2_, which transformed into Fe(OH)_3_. This phenomenon is likely related to the orientation of the samples during the long-term test. The analyzed samples were tilted relative to the liquid level in the test vessel, causing the corrosion products formed on the surface to move gravitationally down the sample and settle primarily at the lowest geometric point, causing localized deposit accumulation and, consequently, the observed visual blackening. It should not be mistakenly concluded that corrosion processes were accelerated locally, as, on the contrary, this is a secondary effect of the migration of reaction products, typical of this sample orientation during the immersion test. The presence of different colors of corrosion products on the observed surfaces is also a result of varying oxygen concentrations in the solution.

At room temperature (21 °C), all samples showed signs of surface degradation, but these were relatively mild and superficial. Unpolished samples were characterized by a distinct dullness and irregular texture.

In the case of samples produced with lower laser power (80 W), whose surfaces are more porous and rough and more susceptible to corrosion, an intensification of corrosion product deposition can be observed. The initiation of larger corrosion damage on the surface, such as pitting and irregularities in the texture continuity, is also visible. The color of corrosion products deposited on samples produced with lower laser power (80 W), especially on polished surfaces, is darker than in samples produced with higher laser power (120 W). Therefore, it can be concluded that the use of lower laser power in the additive manufacturing process results in a greater susceptibility to increased corrosion rates on the surface of such a material at lower environmental temperatures. This is confirmed by the results of the corrosion mass and rate measurements, presented in Table 3.

Increasing the test temperature, in turn, resulted in a more uniform and intense deposition of corrosion products in the observed area. Although differences in the smoothness of polished and unpolished surfaces are visible to the naked eye, macroscopic observation alone reveals that the polished surface is not devoid of roughness. However, the surface irregularities differ in nature from those observed on unprocessed samples: they are regular, repetitive, parallel to each other, and arranged along the sample surface. This substrate creates favorable conditions for the even and uniform deposition of corrosion products throughout the entire area exposed to the aggressive environment. Simultaneously, the increased surface smoothness, along with the presence of individual protrusions and irregularities caused by the migration of unmelted powder residues, facilitates the gravitational slide of corrosion products and their accumulation around the resulting rough geometric points.

The uniformity of corrosion product deposition at higher temperatures is confirmed by the appearance of samples produced at laser powers of 100 W and 120 W and polished, which were better melted during the production process and retained a higher degree of internal structural continuity with limited porosity. As a result, the extent of corrosion product deposition on these samples is greater than on the other analyzed surfaces, and the observed effect is more uniform, both in terms of the deposition size and the color of the corrosion products. It can be assumed that the rate of point corrosion in micro-areas was similar to the average rate of the entire sample surface.

Identification of the colors of the chemical compounds formed as a result of the corrosion reactions suggests that the progress of the electrochemical reactions was uniform throughout the sample, causing the deposit to acquire a black color, characteristic of magnetite Fe_3_O_4_, which has a structure of mixed Fe(II) and Fe(III) oxidation states. Greenish or greenish-black colors are also visible on the surfaces of some samples, indicating the presence of ferrous hydroxide Fe(OH)_2_, which is characteristic of the early stages of the corrosion process. This indicates that oxidation occurred more slowly in the given area than in areas with darker deposits, or that the oxygen concentration in this area of the solution was lower. The presence of red colors, particularly visible on unpolished surfaces, suggests the presence of Fe_2_O_3_, in which iron occurs in the oxidized Fe(III)^+^ state.

It is worth noting that when comparing polished samples subjected to corrosion at elevated temperatures, the surfaces produced at 80 W showed poorer, non-uniform coverage by corrosion products than the 100 W and 120 W samples. Despite polishing, the material produced at 80 W exhibited an increased risk of discontinuities and increased surface roughness compared to the 100 W and 120 W samples due to poorer powder fusion. The protrusions thus formed on the material’s surface can serve to accumulate deposited corrosion products, while local structural discontinuities, gaps, and material voids promote the formation of local corrosion cells, with increased corrosion rates in the micro-area. As part of the potentiodynamic polarization study, the influence of two technological factors on the corrosion resistance of M350 tool steel was analyzed:

- Laser power used during sample production (80 W, 100 W, and 120 W);
- Surface treatment (unpolished and mechanically polished samples).

**Figure 6:**
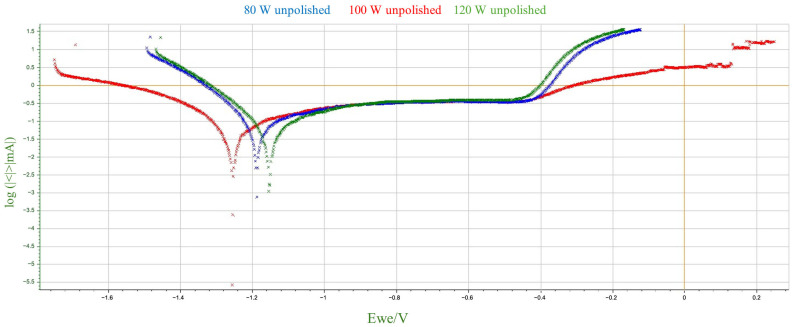
Potentiodynamic polarization curves in 3.5% NaCl solution for unpolished M350 maraging steel samples produced by the LPBF method.

**Figure 7:**
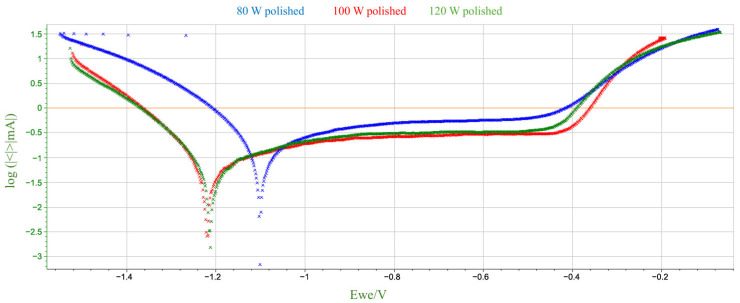
Potentiodynamic polarization curves in 3.5% NaCl solution for polished M350 maraging steel samples produced by the LPBF method.

The parameters used to assess corrosion resistance were: corrosion potential (Ecorr), which determines the thermodynamic tendency of the material to corrode, and corrosion current (Icorr), which determines the rate of the corrosion process. The summary results of the electrochemical parameter measurements are presented in Table 6 and Figure 6 and Figure 7, broken down by mechanical treatment of surface.

Based on the above data, a number of changes in corrosion potential values can be observed as a function of laser power and surface treatment. For unpolished samples, there is no monotonic linear relationship between E_corr_ and laser power. Corrosion potential reaches its lowest value at 100 W, and higher values at 80 W and 120 W. For polished samples, Ecorr values fall within a narrower range, although the 80 W sample was noticeably higher than the others. The differences between polished and unpolished samples for the same power are not unidirectional—for 80 W and 120 W, corrosion potential values are higher for polished samples, while for 100 W, they are lower. Mechanical polishing at low power (80 W) appears to increase the corrosion potential, which may suggest a lower susceptibility to corrosion initiation. This, in turn, contradicts the very high corrosion current in the same sample. For higher laser powers, the differences are marginal. Therefore, it can be concluded that, within the analyzed parameters, there is no clear correlation between the corrosion potential value and either laser power or surface treatment method. The variability in the observed values may indicate the influence of several coexisting technological and microstructural factors.

A comparison of the data obtained during the potentiodynamic polarization test indicates a clear decreasing trend in the corrosion current value with increasing laser power for unpolished samples. No similar clear trend is observed for polished samples. For the sample produced at 80 W, polishing led to a significant increase in the corrosion current value to 237.02 µA—more than twice that of the unpolished sample. This significant increase in corrosion rate suggests that the removal of the surface layer by mechanical processing may have exposed microstructural defects or discontinuities that were previously camouflaged by the irregular, heterogeneous surface topography obtained by the LPBF process. It can be assumed that at low laser power, the sample structure contained more unmelted fragments or pores just below the surface, which, after polishing, could become active sites for pitting corrosion initiation. However, for samples produced at higher laser powers (100 W and 120 W), polishing did not significantly affect the corrosion current, which remained comparable to unpolished samples. It can be assumed that at higher additive manufacturing parameters, the surface was already sufficiently homogeneous despite the lack of mechanical processing, and polishing did not expose additional defects that could increase susceptibility to corrosion.

**Figure 8:**
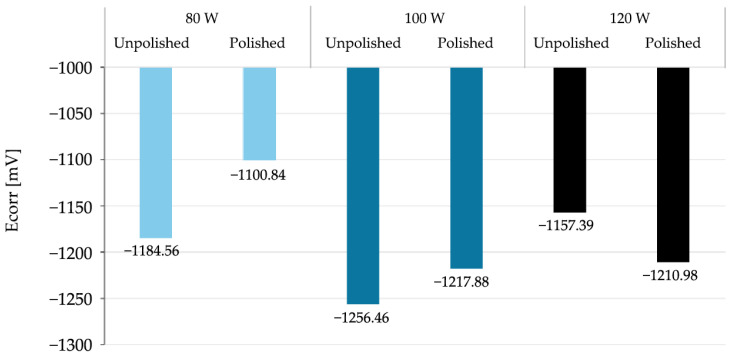
Graph of the dependence of corrosion potential on the surface development state and laser power used during additive manufacturing.

**Figure 9:**
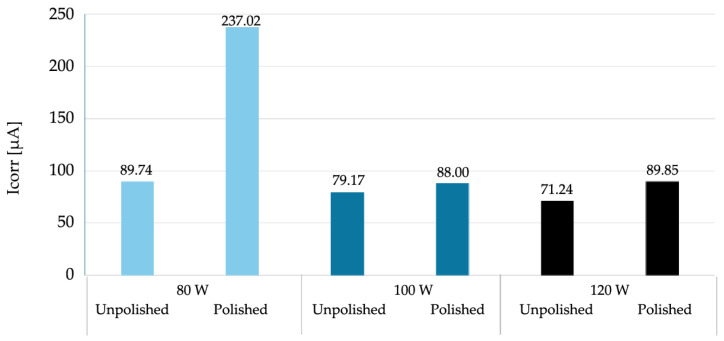
Graph of the dependence of corrosion current on the surface development state and laser power used during additive manufacturing.

In summary, the obtained results, presented collectively in Figure 8 and Figure 9, correspond to the commonly accepted observation regarding the relationship between additive manufacturing parameters and the corrosion susceptibility of materials produced using additive technologies. For unpolished materials, higher laser power can lead to better powder fusion, reduced porosity and potential microcracks, and a more uniform microstructure, resulting in improved corrosion resistance. This phenomenon can be attributed to the higher energy density introduced into the material during the LPBF process, which at higher power levels promotes more complete fusion of the metal powder. Potentiodynamic polarization tests showed that mechanical treatment alone does not clearly improve corrosion resistance; its effect depends on the quality of the structure created during the additive manufacturing process. At low power, polishing could expose surface defects that were not present in the as-prepared state. From a corrosion resistance perspective, this is associated with the discovery of local anodic defects on the sample surface, such as closed pores or oxides. The described structure promotes the destabilization of the passive layer and the development of galvanic microcells, which in turn results in a higher corrosion rate in the NaCl environment.

Table 7 presents the results of optical microscopy observations of the structure of M350 maraging steel samples before and after corrosion at room and elevated temperatures at 100× magnification. The surfaces of samples that were not mechanically polished, prior to corrosion, clearly show the laser beam scan lines, as well as micro-steps between these lines. The polished surfaces, on the other hand, reveal parallel, continuous polishing grooves—precise traces of the machining tool’s passage. Small inclusions, powder residues from the additive manufacturing process, are also visible in the observed area. Polishing removed the rough laser powder bed fusion scan lines but introduced a distinct directional texture, which could constitute potential fatigue crack initiation paths or corrosion cells. A similar picture is presented in the scanning electron microscope images in Table 8. For unpolished surfaces, a 1000× zoom does not clearly reveal the laser paths, but surface irregularities typical of the upskin area after printing are visible.

The long-term low-temperature corrosion results in a visible dulling of the surface of the observed samples. In the case of the unpolished surface, corrosion products partially fill the depressions and gaps between the laser paths. The depth of the rough depressions decreases due to the slow growth of oxides. Similarly, in the case of the polished surface, the observed area was covered with a thin layer of corrosion products, which partially covered the depressions left by the polishing tool. The laser transition lines remained visible, as seen in darker areas in the optical microscope images.

In the case of corrosion at elevated temperatures, the progressive buildup of corrosion products in the form of oxides and hydroxides is visible. The unpolished surface exhibits numerous oxide inclusions, pit initiations, and microcracks. Local smoothing was observed on the polished surface, associated with the degradation of the passive layer, along with the formation of cracks, which occurred less frequently than on the unpolished surface.

With increasing laser power, the tendency for pitting and microcracks to form increases, especially on unprocessed surfaces. Dense, needle-like oxide aggregates develop, as do networks of irregular pores and secondary cracks and crevices. Local spherical powder residues become visible.

Although the polished surface is characterized by greater uniformity and allows for the observation of areas of greater continuity than on unprocessed surfaces, this material is not free from cracks, micropores, and potential sources of pitting corrosion. Observation with an optical microscope reveals a cross-section of the laser scan traces after the additive manufacturing process (80 W, low corrosion temperature, polished). Scanning electron microscopy observations also reveal the presence of fine, linear outlines, which are scattered, less visible, and aligned parallel to the polishing groove. These likely represent residual texture after polishing and, at elevated temperatures in a corrosive environment, may constitute corrosion initiation zones, although at a lower intensity than the deeper grooves left by the machining tool. A large crack was observed in the sample area produced at 100 W, after polishing and exposure to corrosion at 45 °C, likely resulting from gas pores in the printed structure. Further cracks radiated from the main crack, suggesting a localized corrosion cell. In this case, polishing smoothed the surface of the sample but did not remove the micropores.

SEM microstructure images, included in Table 7, reveal the presence of numerous cracks on the observed surfaces. The presence of microchannels, created as a result of incomplete melting of the metal powder between the individual layers in the incremental process, and the temperature gradient causing shrinkage and local stresses allowed the electrolyte to penetrate deeper into the samples. The roughness and the presence of these micro-holes result in greater corrosion susceptibility of the samples in the outer layer. In the context of the characteristic crack shapes observed on the polished surface, two mechanisms should be considered, perhaps co-influencing the final structure of the material. The first is the induction of a decrease in local stresses by the corrosion process, which ultimately led to the relaxation of stresses in the material and cracks along the relaxing areas. The second mechanism is the initiation of corrosion along grain boundaries, which would explain the characteristic shape of the observed cracks. The central area of individual grains has different structural parameters than the boundaries of these grains, resulting in corrosion progressing at varying rates depending on the area within the analyzed grains.

These crack formation mechanisms likely coexist—varying structural parameters within the grains lead to different corrosion rates in micro-areas, causing corrosion at the grain boundaries, which simultaneously reduces the stresses that maintain the printed material in its original compact form. The relaxation of stresses and the intensification of corrosion ultimately cause the formation and subsequent expansion of cracks. The deepening discontinuities in the material allow the electrolyte better access to the internal micropores, where further corrosion cells form and the material degradation process progresses.

The EDS detector allowed for determination of the chemical composition of the micro-areas observed under a scanning electron microscope. A representative test area for a given sample was designated on the examined surface. Characteristic points in each of the observed areas were also examined. The chemical composition measurement results for the samples subjected to long-term corrosion testing at room temperature are presented in Table 9, while the results for testing at elevated temperature (45 °C) are presented in Table 10.

The indicated chemical elements detected on the surface of the tested samples match the expected chemical composition of the material. The presence of S and Si was identified. These are contaminants originating from the process coolants and the water used to purify the material. The results reveal an increased presence of carbon, indicating contamination, likely originating from the additive manufacturing process and the formation of a deposit due to the applied laser power and laser burn-through. This is confirmed by the fact that carbon was detected on surfaces produced with the highest laser power (120 W).

Oxygen was also present, a component of oxides and hydroxides resulting from the corrosion process. The percentage of oxygen present on the sample surface changes according to the trend line shown in Figure 3, Figure 4 and Figure 5, confirming that the relationship between the corrosion rate, which was assumed to be an indicator of the process’s progress, and the technological factors of material preparation and the parameters of the corrosive environment is valid and is also reflected in the other obtained results. It is also worth noting the presence of the elements Ni, Mo, Ti, and V, which form various types of precipitates in maraging steels, such as Ni3Mo, Ni3Ti, Laves Fe2Mo, Laves Fe2Ti, σ–FeMo, σ–FeTi, and µ–Fe7Mo6. Increasing the molybdenum content can result in faster kinetics and higher density of Ni3Ti precipitation. The addition of titanium, in turn, influences the formation of finer precipitates rich in molybdenum.

The presence of silicon and sulfur was also detected on the surface—elements that are not present in the chemical composition of the M350 maraging steel powder nor are they a component of the corrosive environment in which the tests were conducted. The presence of silicon can be explained by the use of polishing tools. The sandpaper used contained silicon carbide (SiC), a sharp abrasive material used in the machining of hard surfaces. Due to the high hardness of the M350 maraging steel, up to 650 HV5, it was pressed against the sample surface using high friction forces. Given the porous, rough surface of the samples, and due to the high-temperature properties characteristic of products obtained by the LPBF method, silicon microparticles had excellent conditions for retention in the channels and cracks in the original material exposed during the polishing process. Sulfur is likely a component of the fluid supporting the mechanical polishing process, which was not completely purified by distilled water and ethanol after processing.

**Figure 10:**
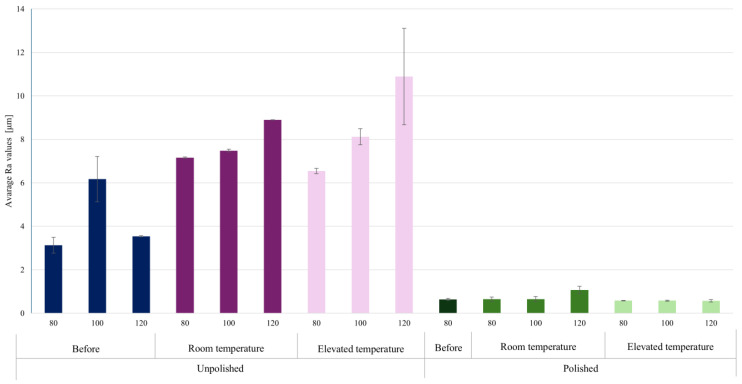
Graph of average Ra values for the tested surfaces depending on the laser power used in the additive manufacturing process, surface treatment, and the temperature of the corrosive environment.

**Figure 11:**
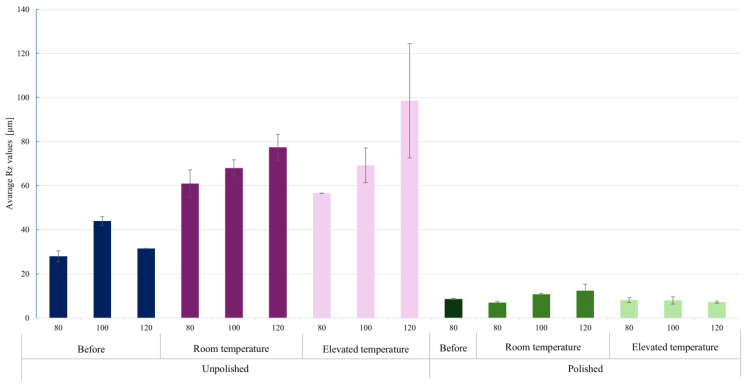
Graph of average Rz values for the tested surfaces depending on the laser power used in the additive manufacturing process, surface treatment, and the temperature of the corrosive environment.

The surfaces of materials produced by the LPBF method are typically rough due to the characteristics of the additive process. This is due to the growing layers of molten powder, uneven solidification of the melt pool, and the adhesion of unmelted metal powder particles. Mechanical polishing is commonly used to reduce roughness and improve surface properties. Table 11 presents the results of roughness measurements performed on a profilometer for 18Ni-350 steel samples. The Ra and Rz parameters were determined for the tested surfaces. Ra (arithmetic mean roughness) is the arithmetic mean of the deviations of the actual profile from the mean line, calculated along a specific measurement section. Rz (average maximum height of the profile) is the arithmetic mean of the heights of the five highest peaks and five deepest depressions within the measurement section. The graph in Figure 10 presents a summary of the average Ra values, while the graph in Figure 11 presents the average Rz values.

The data obtained clearly show that the use of mechanical polishing significantly reduces both the arithmetic mean profile deviation (Ra) and the maximum profile height (Rz) compared to unpolished samples. For unpolished samples, Ra values ranged from 6.54 μm to 10.89 μm, while for polished samples they ranged from 0.57 μm to 1.07 μm. Simultaneously, the Rz parameter ranged from 56.59 μm to 98.49 μm for unpolished surfaces, while for polished samples it did not exceed 12.31 μm. This reduction in roughness confirms the effectiveness of the polishing process in eliminating deep surface defects.

Increasing laser power from 80 W to 120 W resulted in a significant increase in roughness parameters, particularly for unpolished surfaces. The highest Ra (8.90–10.89 μm) and Rz (77.39–98.49 μm) values were recorded at the highest laser power and elevated process temperature. This indicates a strong influence of laser beam energy on the intensification of local remelting and surface defects, which, combined with corrosion, lead to the formation of deeper irregularities. This also confirms the observations regarding the long-term corrosion test results.

Comparing the results obtained at room temperature and elevated temperature (45 °C), a noticeable increase in roughness was observed with increasing temperature for the unpolished samples. For this group of samples, Ra increased from a value of 7–9 μm at low temperature to a maximum value of 10.89 μm at elevated temperature. Similarly, the Rz parameter increased to values close to 100 μm, indicating intensified corrosion phenomena and the formation of deep cavities. For polished samples, the effect of temperature was less significant—the Ra and Rz values remained similar. This corresponds to the results in Table 3, which describes the relationship between the corrosion rate ratio at individual temperatures depending on technological factors during production and surface preparation. Polishing limits the effect of temperature on the progression of corrosion processes.

**Figure 12:**
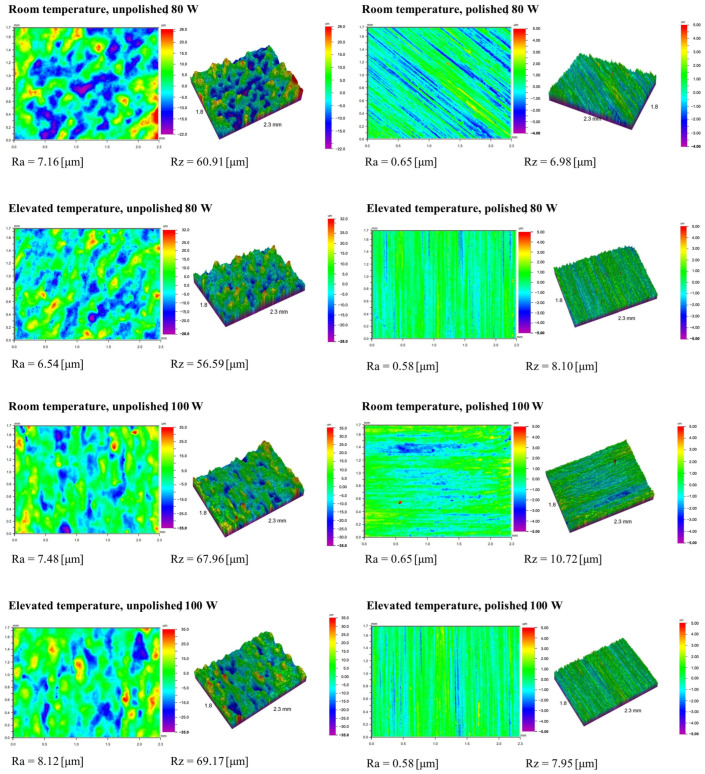

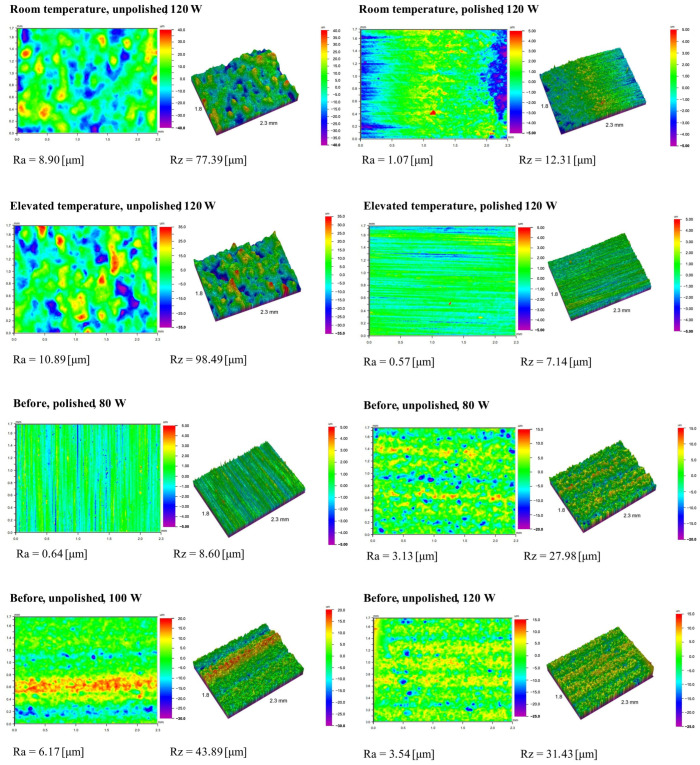
Surface topography of samples subjected to roughness testing.

The two- and three-dimensional images obtained after the roughness test, presented in the summary table in Figure 12, demonstrate the uniformity of surface conditions depending on the mechanical treatment. The left side of the table shows unpolished surfaces: their profile is more diverse, with irregular, yet predictable, surface irregularities and numerous depressions in areas that likely initiated corrosion. The samples on the right side of the table represent materials that were polished: typical polishing tool marks, depressions, and lines previously observed under optical and scanning microscopes are visible. The results obtained using each test method complement each other. An interesting observation is that for the highest laser power (120 W), polishing allowed for a nearly twenty-fold reduction in the Ra parameter. At the same time, Table 3 indicates that polishing alone did not effectively reduce the corrosion rate at any of the long-term test temperatures. The high corrosion rate and progression of this process, and consequently the intense deposition of corrosion products, could have led to the filling of polishing grooves and a smoothing of the surface, which thus increased its smoothness.

## 4. Conclusions

The conducted studies showed that temperature, surface treatment, and additive printing parameters have a significant impact on the corrosion resistance of M350 maraging steel. The following conclusions can be drawn:

1. For operation at room temperature, high laser power (≥120 W) is optimal, while for operation at elevated temperatures, such as 45 °C, lower laser power (80–100 W) is preferable.
2. At room temperature, higher laser power (120 W) reduced the corrosion rate, while at 45 °C, the trend was reversed, with the highest power generating the greatest mass loss.
3. Mechanical polishing did not improve resistance; on the contrary, in several systems, it increased the corrosion rate, which can be attributed to the relocation of residual stresses and the exposure of surface defects. Mechanical polishing should be combined with stress reduction methods, e.g., aging, and porosity reduction, e.g., hot isostatic pressing.
4. The rate of corrosion reaction acceleration with temperature (45 °C/8–24 °C) increased with laser power, while mechanical polishing reduced it only for manufacturing powers of 80 W and 120 W. Temperature sensitivity can be reduced by minimizing porosity and controlling the microstructure during the additive manufacturing stage using the laser powder bed fusion method.
5. Potentiodynamic polarization tests revealed that the effectiveness of polishing as a treatment for improving corrosion resistance is strongly dependent on the original surface quality, which in turn depends on the LPBF process parameters. For surfaces fabricated with low laser power (80 W), polishing resulted in a significant deterioration in resistance: I_corr_ increased by more than 2.5-fold, even though E_corr_ shifted towards a less negative value. For 100 W and 120 W, surface treatment affected I_corr_ less, and the differences from the as-received state were small—a change of approximately 10 μA. Electrochemical resistance increased with laser power as long as the surface remained as-received, but this effect disappeared after aggressive polishing.
6. After the fabrication process, unpolished surfaces were characterized by distinct laser scan lines and the presence of micro-steps, which were removed by polishing. However, mechanical treatment left parallel grooves, introducing a directional texture on the surface that could easily become a path for crack propagation, posing a risk of pitting corrosion.
7. Higher laser power leads to increased roughness, which may affect the corrosion and mechanical properties of the material.

## Figures and Tables

**Table 1:** Chemical composition of the powder used to produce the test samples [43].

Chemical Composition of the Powder								
Element	Fe	C	Ni	Co	Mo	Ti	V	Al
Mass percentage (%)	Balance	<0.1	<20	<12	<5	<2	<2	<0.5

**Table 2:** Technical parameters of the SLM280 printer from Nikon SLM Solutions AG used to produce the test samples.

Laser spot diameter	85 µm
Build plate temperature	200 °C
Laser power	Max. 250 W
Scanning speed	Max. 850 mm/s
Scanning increment	100 µm

**Table 3:** Mass measurement results before and after long-term corrosion testing and corrosion rate values for individual groups of M350 steel samples.

Sample Surface Treatment	Temperature	Laser Power (W)	Average Mass Difference (g)	Average Mass Loss (%)	Average Corrosion Rate (g/cm^2^ × year)	Corrosion Rate Standard Deviation (g/cm^2^ × year)
Raw	Room (21 °C)	80	0.0064	0.2302	44.69	8.19
		100	0.0059	0.2107	41.33	5.80
		120	0.0059	0.2182	41.39	3.40
	High (45 °C)	80	0.0122	0.4421	85.90	2.53
		100	0.0125	0.4485	87.51	0.89
		120	0.0131	0.4820	91.62	3.40
Mechanically Polished	Room (21 °C)	80	0.0065	0.3047	45.37	7.64
		100	0.0061	0.2840	42.56	7.58
		120	0.0061	0.2884	42.61	5.66
	High (45 °C)	80	0.0113	0.5246	79.48	4.81
		100	0.0129	0.6031	90.38	3.06
		120	0.0128	0.6005	89.49	6.30

**Table 4:** Comparison of trend line slope values depending on corrosion temperature and surface treatment application.

Temperature	Surface Treatment	Slope Coefficient
Room (21 °C)	Unpolished	−1.65
	Polished	−1.38
High (45 °C)	Unpolished	2.86
	Polished	5.01

**Table 5:** Results of macroscopic surface analysis after long-term corrosion testing.

Surface Treatment	Laser Power	Before Long-Term Corrosion Test	After Long-Term Corrosion Test	
			Room Temperature (21 °C)	Elevated Temperature (45 °C)
Unpolished	80 W	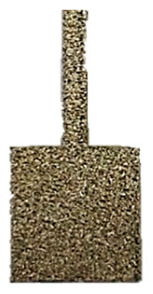	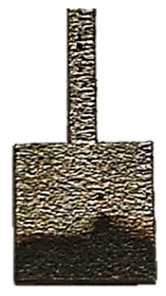	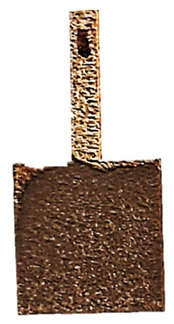
	100 W	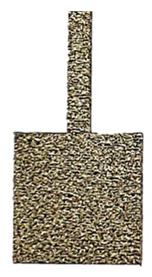	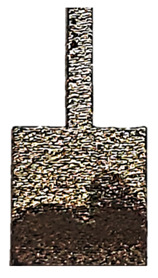	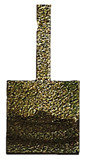
	120 W	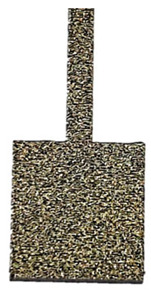	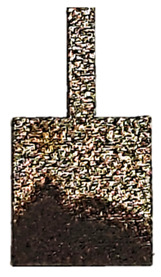	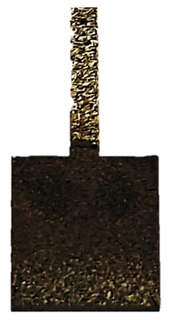
Polished	80 W	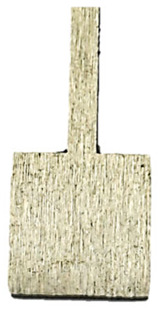	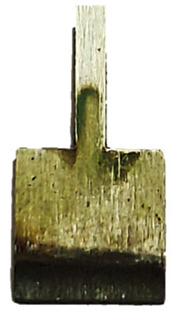	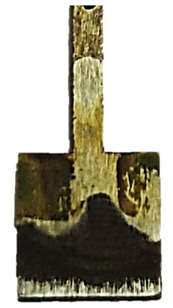
	100 W	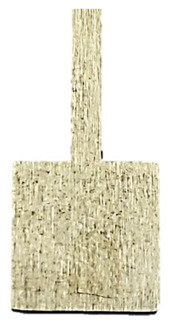	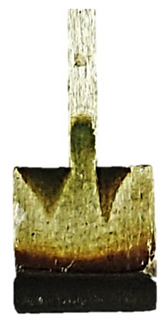	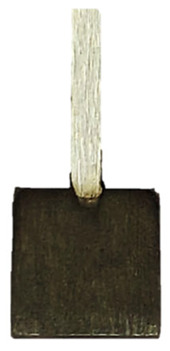
	120 W	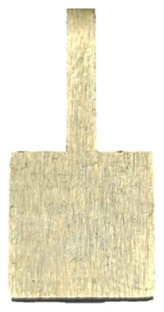	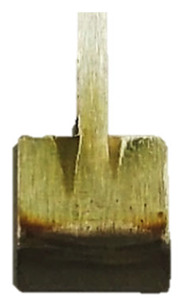	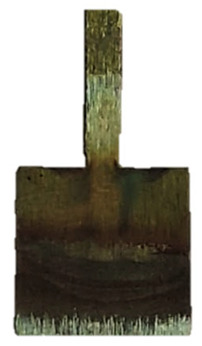

**Table 6:** Potentiodynamic polarization test results.

Surface Treatment	Laser Power [W]	E_corr_ [mV]	I_corr_ [μA]
Unpolished	80	−1184.56	89.74
	100	−1256.46	79.17
	120	−1157.39	71.24
Polished	80	−1100.84	237.02
	100	−1217.88	88.00
	120	−1210.98	89.85

**Table 7:** Images of the structure of unpolished and polished surfaces, produced with laser power of 80 W, 100 W, and 120 W, respectively, before and after the corrosion process at room and high temperature, taken on an optical microscope with ×100 magnification.

Unpolished			
Laser Power	Before Corrosion Test	After Corrosion in Room Temperature	After Corrosion in Elevated Temperature
80 W	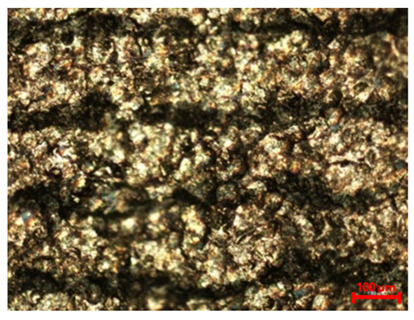	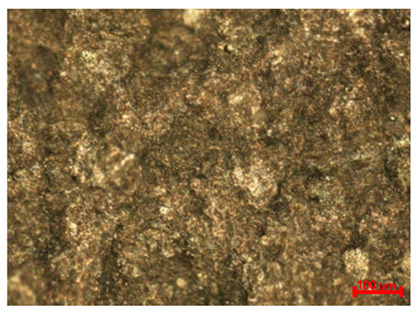	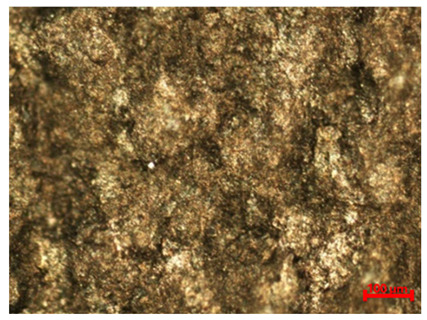
100 W	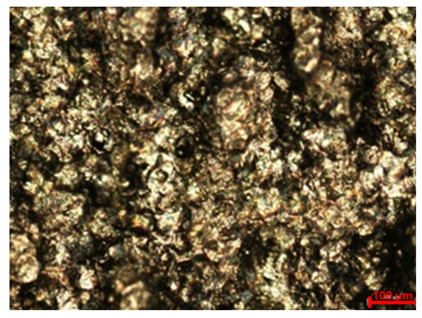	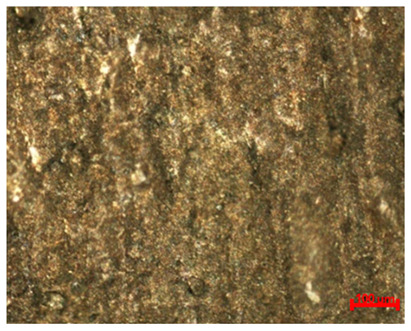	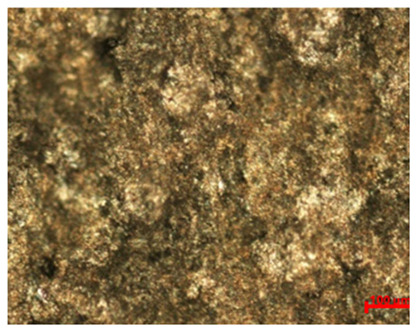
120 W	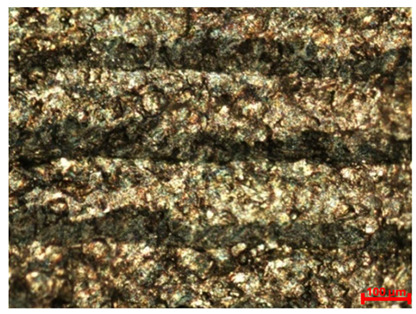	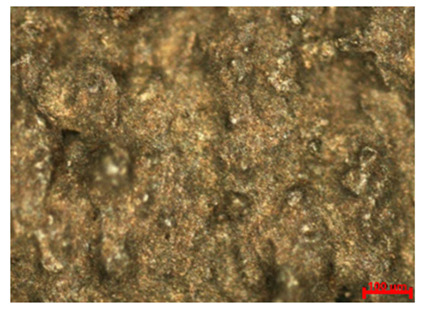	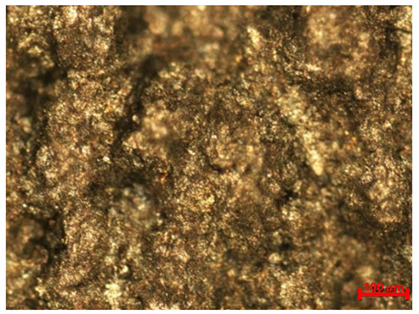
**Polished**			
**Laser Power**	**Before Corrosion Test**	**After Corrosion in Room Temperature**	**After Corrosion in Elevated Temperature**
80 W	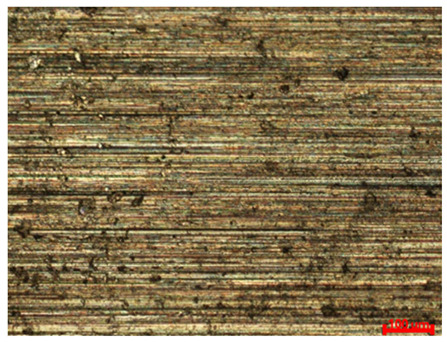	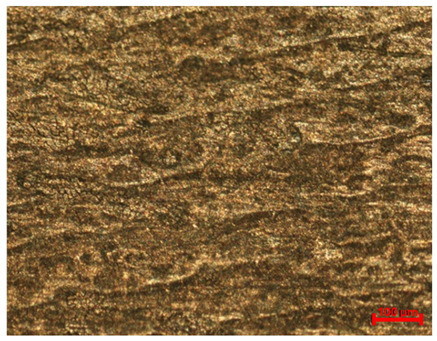	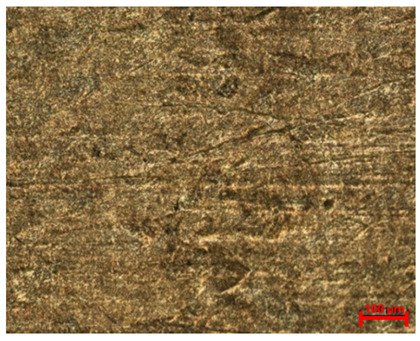
100 W	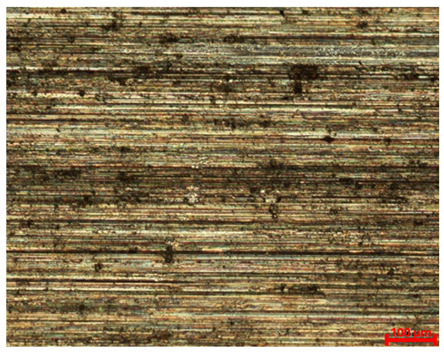	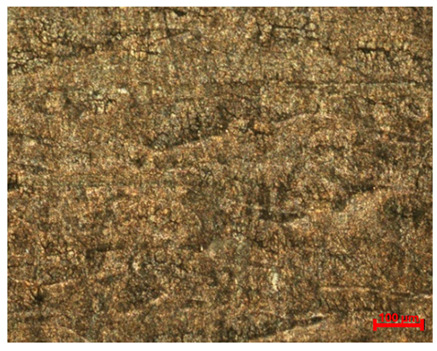	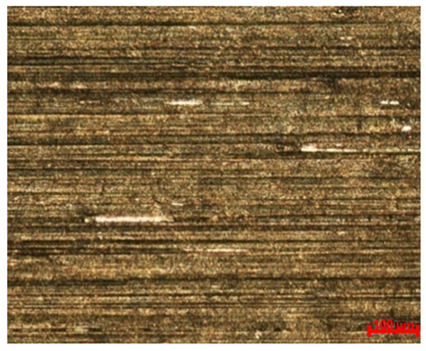
120 W	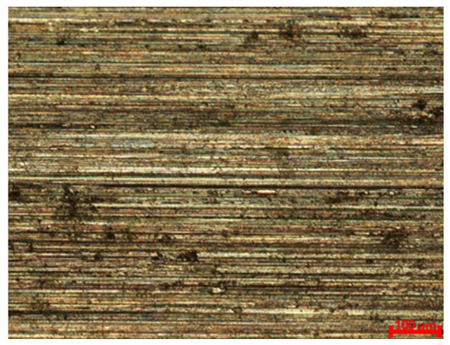	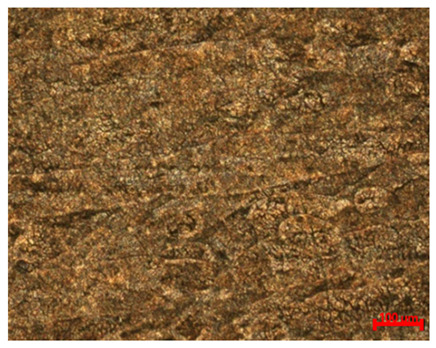	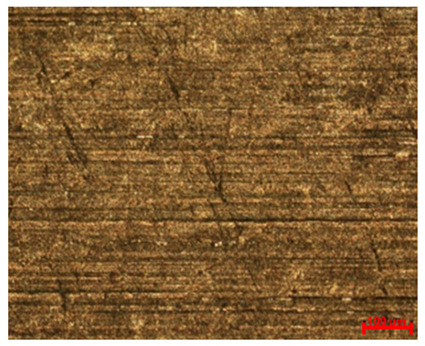

**Table 8:** Images of the structure of unpolished and polished surfaces, produced with a laser power of 80 W, 100 W, and 120 W, respectively, before and after the corrosion process at room and high temperature, taken on a scanning microscope with a magnification of ×1000.

Unpolished			
Laser Power	Before Corrosion Test	After Corrosion in Room Temperature	After Corrosion in Elevated Temperature
80 W	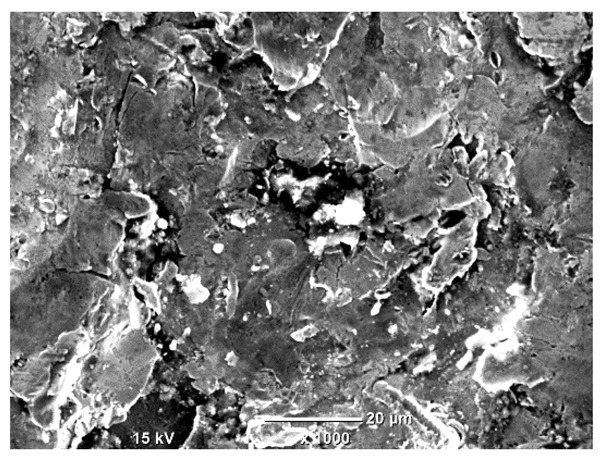	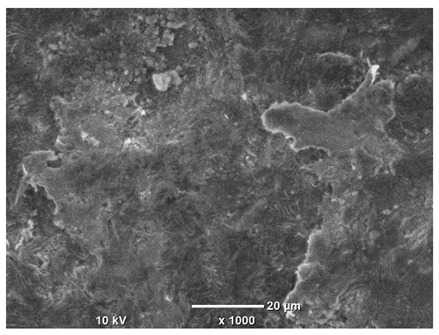	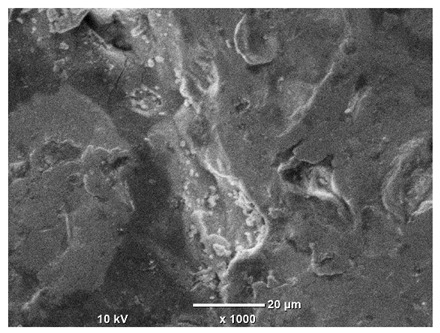
100 W	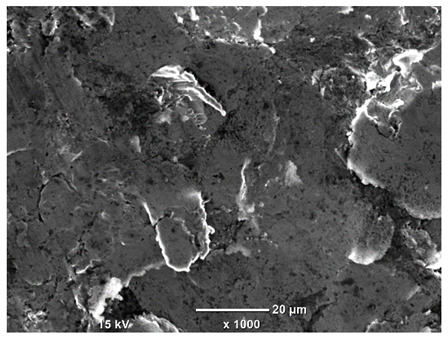	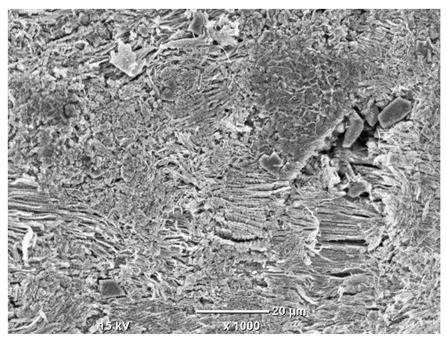	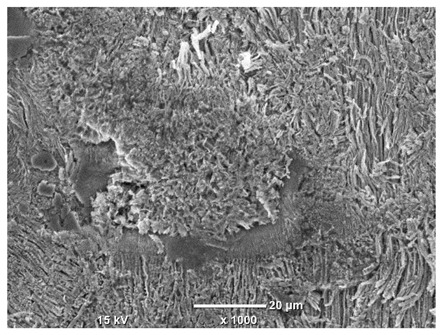
120 W	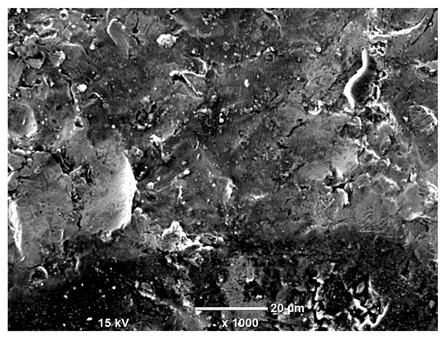	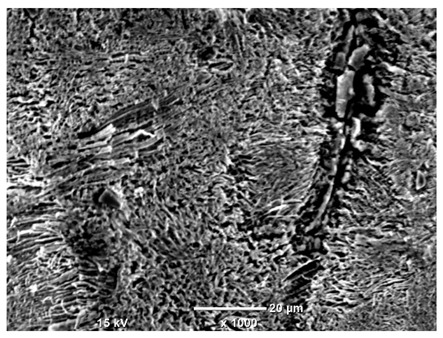	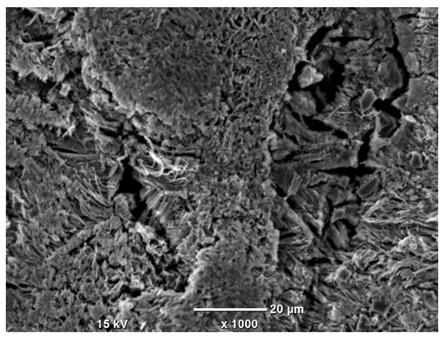
**Polished**			
**Laser Power**	**Before Corrosion Test**	**After Corrosion in Room Temperature**	**After Corrosion in Elevated Temperature**
80 W	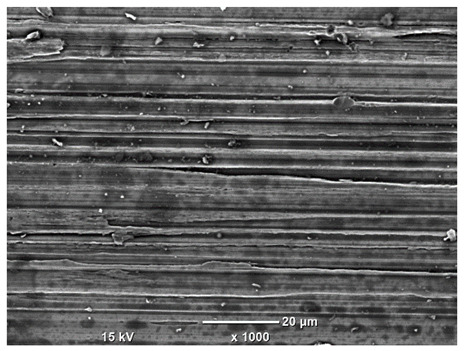	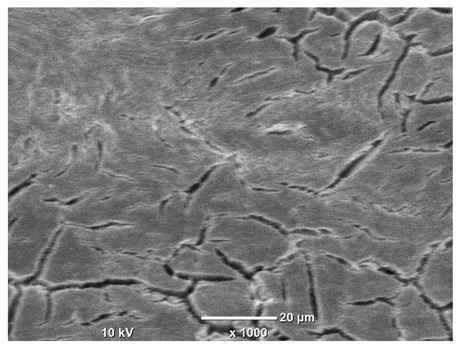	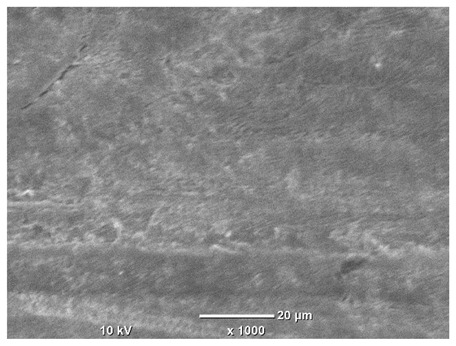
100 W	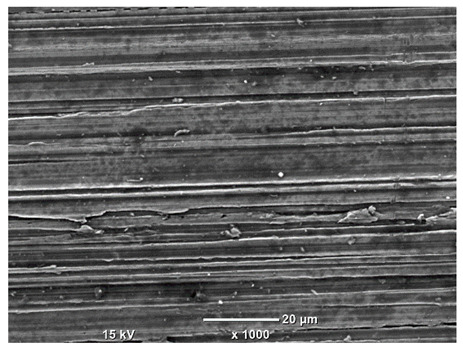	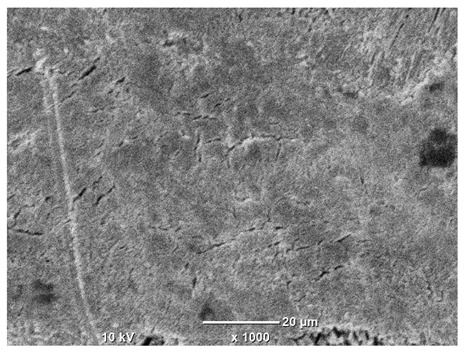	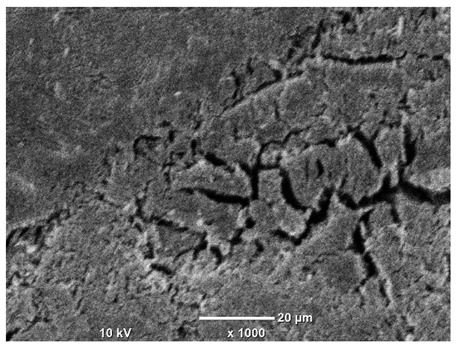
120 W	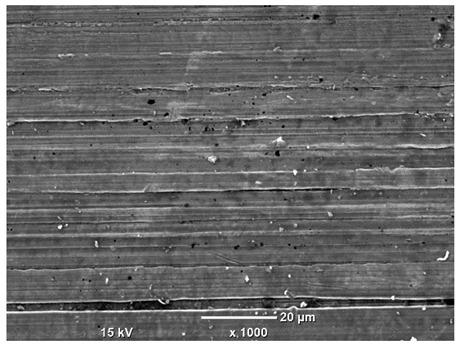	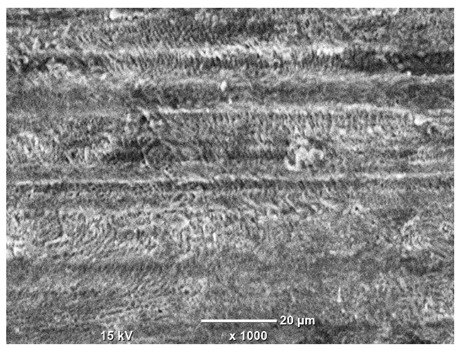	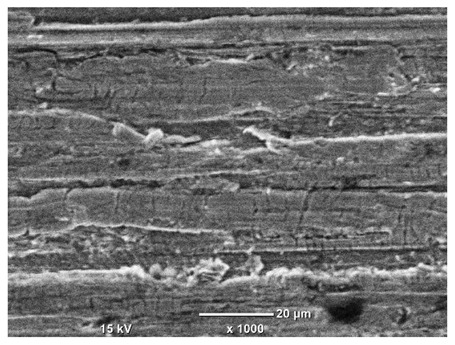

**Table 9:** Chemical composition analysis results for surfaces subjected to long-term corrosion testing at room temperature.

Treatment	Laser Power (W)	%	Al	C	Fe	O	S	Si	Ti	V	Ni	Mo
Unpolished	80	Mass	0.71	x	40.94	20.13	0.47	0.41	5.09	2.14	24.42	5.69
	100	Mass	0.74	x	48.82	13.38	0.79	0.90	5.34	4.19	18.82	7.02
	120	Mass	0.76	3.67	40.67	17.77	1.22	0.95	6.25	6.71	13.78	8.20
Polished	80	Mass	0.98	x	28.74	21.28	2.81	0.94	8.48	10.72	14.78	11.27
	100	Mass	0.96	x	43.95	14.69	1.82	0.84	3.47	5.12	19.46	9.69
	120	Mass	0.53	x	48.77	17.51	0.71	0.67	3.19	5.48	16.97	6.17

**Table 10:** Chemical composition analysis results for surfaces subjected to long-term corrosion testing at elevated temperatures.

Treatment	Laser Power (W)	%	Al	C	Fe	O	S	Si	Ti	V	Ni	Mo
Unpolished	80	Mass	2.38	x	47.29	14.87	x	0.72	12.04	0.47	18.54	3.68
	100	Mass	1.04	x	40.55	22.34	1.07	0.69	6.08	7.40	13.45	7.38
	120	Mass	0.85	2.42	37.80	19.55	1.54	0.74	7.25	8.68	11.63	9.54
Polished	80	Mass	x	x	40.80	14.77	1.84	0.67	6.00	6.31	21.78	7.83
	100	Mass	0.27	x	44.76	13.87	1.45	0.38	5.42	8.51	17.97	7.37
	120	Mass	0.20	6.20	54.60	10.25	1.04	0.51	2.64	2.93	17.99	3.64

**Table 11:** Ra and Rz measurement results for the tested surfaces.

Surface Treatment	Corrosion Temperature	Laser Power (W)	Ra (μm)	Rz (μm)
Unpolished	Before Corrosion	80	3.13	27.98
		100	6.17	43.89
		120	3.54	31.43
	Room (21 °C)	80	7.16	60.91
		100	7.48	67.96
		120	8.90	77.39
	Elevated (45 °C)	80	6.54	56.59
		100	8.12	69.17
		120	10.89	98.49
Polished	Before Corrosion	80	0.64	8.60
	Room (21 °C)	80	0.65	6.98
		100	0.65	10.72
		120	1.07	12.31
	Elevated (45 °C)	80	0.58	8.10
		100	0.58	7.95
		120	0.57	7.14

## Data Availability

The original contributions presented in this study are included in the article. Further inquiries can be directed to the corresponding author.
